# Darkness and *gulliver2*/*phyB* mutation decrease the abundance of phosphorylated BZR1 to activate brassinosteroid signaling in Arabidopsis

**DOI:** 10.1111/tpj.12423

**Published:** 2014-02-04

**Authors:** Bokyung Kim, Yu Jeong Jeong, Claudia Corvalán, Shozo Fujioka, Seoae Cho, Taesung Park, Sunghwa Choe

**Affiliations:** 1 School of Biological Sciences, College of Natural Sciences, Seoul National UniversitySeoul, 151-747, Korea; 2 RIKEN Advanced Science InstituteWako-shi, Saitama, 351-0198, Japan; 3 Interdisciplinary Program in Bioinformatics, College of Natural Science, Seoul National UniversitySeoul, 151-747, Korea; 4 Department of Statistics, College of Natural Sciences, Seoul National UniversitySeoul, 151-747, Korea; 5 Plant Genomics and Breeding Institute, Seoul National UniversitySeoul, 151-921, Korea; 6 Convergence Research Center for Functional Plant Products, Advanced Institutes of Convergence TechnologyGwanggyo-ro 145, Yeongtong-gu, Suwon-si, Gyeonggi-do, 443-270, Korea

**Keywords:** brassinosteroids, photomorphogenesis, hypocotyl elongation, proteasome, brassinazole, *Arabidopsis thaliana*

## Abstract

Light is essential for plant survival; as such, plants flexibly adjust their growth and development to best harvest light energy. Brassinosteroids (BRs), plant growth-promoting steroid hormones, are essential for this plasticity of development. However, the precise mechanisms underlying BR-mediated growth under different light conditions remain largely unknown. Here, we show that darkness increases the activity of the BR-specific transcription factor, BZR1, by decreasing the phosphorylated (inactive) form of BZR1 in a proteasome-dependent manner. We observed that COP1, a dark-activated ubiquitin ligase, captures and degrades the inactive form of BZR1. In support of this, BZR1 is abundant in the *cop1-4* mutant. The removal of phosphorylated BZR1 in darkness increases the ratio of dephosphorylated to phosphorylated forms of BZR1, thus increasing the chance of active homodimers forming between dephosphorylated BZR1 proteins. Furthermore, a transcriptome analysis revealed the identity of genes that are likely to contribute to the differential growth of hypocotyls in light conditions. Transgenic misexpression of three genes under the *35S* promoter in light conditions resulted in elongated petioles and hypocotyls. Our results suggest that light conditions directly control BR signaling by modulating BZR1 stability, and consequently by establishing light-dependent patterns of hypocotyl growth in Arabidopsis.

## INTRODUCTION

The ability of plants to adapt their growth patterns to different light conditions underscores an important survival strategy in natural environments. When not exposed to optimal light, plants mobilize resources to grow and reach the light as part of a developmental process termed the shade avoidance syndrome (SAS) (Morelli and Ruberti, 2002; Franklin and Whitelam, 2005; Vandenbussche *et al.*, 2005). Arabidopsis seedlings exhibiting SAS have longer hypocotyls and smaller leaf blades than their light-grown counterparts. Growth-promoting plant hormones, including auxin and gibberellins, control SAS (Pierik *et al.*, 2004; Alabadi *et al.*, 2008; Feng *et al.*, 2008; de Lucas *et al.*, 2008; Tao *et al.*, 2008; Stavang *et al.*, 2009). Many genes are known to be involved in SAS (Carabelli *et al.*, 1996; Steindler *et al.*, 1999; Kozuka *et al.*, 2005; Bou-Torrent *et al.*, 2008; Sorin *et al.*, 2009; Leivar and Quail, 2011). Arabidopsis loss-of-function *phytochrome B* (*phyB*) mutants (Bae and Choi, 2008) and *PHYTOCHROME INTERACTING FACTOR* (*PIF*) overexpression lines constitutively display SAS in the light (Kim *et al.*, 2003), suggesting that these genes control the growth pattern under various light conditions.

Brassinosteroids (BRs) are growth-promoting plant steroid hormones, and thus mutants defective in BR biosynthesis or signaling display characteristic growth-deficiency phenotypes (Clouse *et al.*, 1996; Li *et al.*, 1996; Szekeres *et al.*, 1996; Choe *et al.*, 1998, 1999a,b, 2000). Interestingly, SAS phenotypes of *phyB* disappear when combined with mutations defective in BR biosynthesis (Luccioni *et al.*, 2002), suggesting that BRs are implicated in *phyB-*mediated hypocotyl growth. However, the mechanism by which light conditions control the degree of BR activity remains elusive. A current model depends on the interplay of the two transcription factors: PIF4 and BRASSINAZOLE RESISTANT 1 (BZR1) (Oh *et al.*, 2012). When seedlings elongate in darkness, PIF4 and BZR1 form a heterodimer that regulates the transcription of their target genes; *GERMIN-LIKE PROTEIN 1* (*GER1*) is repressed and *PACLOBUTRAZOL RESISTANCE* genes (*PRE5* and *PRE6*) are activated (Oh *et al.*, 2012). As PIF4 is a light-labile protein (Lorrain *et al.*, 2008), BZR1 stimulates growth more effectively in darkness. This model is also supported by the phenotype of the gain-of-function mutant, *bzr1-D*, which shows resistance to a BR biosynthetic inhibitor only in darkness (Wang *et al.*, 2002). It is likely that the deficiency of PIF4 in light conditions limits the growth-promoting effect of *bzr1-D*. As such, BRs seem to participate in the differential growth of hypocotyls in part through their interaction with PIF4. However, it is not known how the activity of BZR1 itself is differentially controlled in light and darkness.

BZR1 is a substrate of a glycogen synthase kinase 3β-like kinase, BR INSENSITIVE 2 (BIN2; Choe *et al.*, 2002; Li and Nam, 2002; Li *et al.*, 2001; Perez-Perez *et al.*, 2002). Upon phosphorylation, BZR1 is captured by 14*-*3*-*3 proteins and sequestered in the cytoplasm (Gampala *et al.*, 2007; Ryu *et al.*, 2007), or its DNA binding affinity is reduced as part of an inactivation mechanism (Vert and Chory, 2006). Thus, the ratio of dephosphorylated to phosphorylated BZR1 serves as an index to estimate the signaling status of the plant (Wang *et al.*, 2002). The phosphorylation status of BZR1 does not seem to change under different light conditions (He *et al.*, 2002; Wang *et al.*, 2002), however, and the hypocotyls of the quadruple mutant of *pif1-4* (*pifq*) still elongate in response to darkness (Shin *et al.*, 2009; Leivar and Quail, 2011), suggesting that a mechanism that directly regulates BZR1 activity exists and remains to be identified.

During our quest to discover the mechanisms that govern BZR1 activity, we found that the *gulliver2*/*phyB-77* mutant possesses a reduced level of phosphorylated BZR1 in light. Surprisingly, we found that when plants are transferred from light to darkness, the protein levels of BZR1, mostly the phosphorylated forms, are gradually degraded in a proteasome-dependent manner. *In vivo* pull-down analyses revealed that CONSTITUTIVE PHOTOMORPHOGENIC 1 (COP1), an E3 ligase, binds to phosphorylated forms of BZR1. In addition, transcriptome analyses of *phyB-77*, *bri1-5* and the *phyB-77 bri1-5* double mutant revealed the unique set of genes that is likely to be regulated by BZR1 under the direction of both BR and light signaling. We show that light and darkness directly control components of the BR signaling pathway to confer optimal growth patterns according to the specific light conditions.

## RESULTS AND DISCUSSION

### Upregulation of feedback downregulation signaling in the *phytochrome B*-77 mutant

To understand the molecular mechanisms underlying the interaction between BRs and light, we isolated *gulliver 2* (*gul2*) mutants by screening Arabidopsis mutant populations for elongated hypocotyls in the presence of both a BR biosynthetic inhibitor, brassinazole (Brz), and light. We isolated two alleles: *gul2-1* and *gul2-2*. The *gul2-1* mutant was in the Wassilewskija*-*2 (Ws*-*2) background and was mutagenized by ethyl methanesulfonate (EMS), whereas *gul2-2* was obtained from a T*-*DNA activation-tagged mutant population in the Columbia*-*0 (Col*-*0) ecotype (Kim *et al.*, 2014). Through the combined methods of map-based cloning and candidate gene sequencing, we revealed that both *gul2-1* and *gul2-2* mutants have defects in *PHYTOCHROME B* (*PhyB*), so they were renamed as *phyB-77* and *phyB-78*, respectively. The *phyB-77*/*gul2-1* mutant had a single base-pair substitution that changed Gln at the 438th residue to a premature stop codon. The untagged *phyB-78*/*gul2-2* allele had a deletion mutation at 582–613 bp from the start codon, which caused a frameshift mutation, eventually introducing a premature stop codon at amino acid 196.

**Figure 1:**
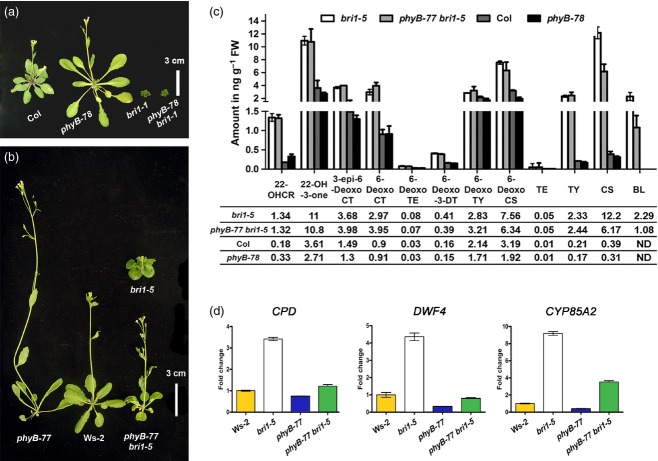
Activation of brassinosteroid (BR) signaling in a weak *bri1-5* allele by the *phyB* mutation. (a) A strong *bri1-1* allele is epistatic to *phyB-78*. (b) A weak *bri1-5* allele is suppressed by *phyB-77*. The dwarfism of *bri1-5* was suppressed by *phyB-77*; the double mutant and the wild type were similar in stature. The plants were grown for 6 weeks under long-day conditions (16*-*h light/8*-*h dark), with a photon fluence rate of 80 μmol m^−2^ s^−1^ at 22°C. (c) Differences in BR levels in 5*-*week-old wild type, *bri1-5*, *phyB-77* and *phyB-77 bri1-5* plants. Tissues were collected from aerial parts. Mean values of up to four biological replicates are shown, with standard errors. Abbreviations: 22-OHCR, 22*-*hydroxycampesterol; 22-OH-3-one, 22-hydroxy-5α-ergostan-3-one; 3-*epi*-6-DeoxoCT, 3-*epi*-6-deoxocathasterone; 6*-*DeoxoCT, 6-deoxocathasterone; 6*-*DeoxoTE, 6*-*deoxoteasterone; 6*-*Deoxo3DT, 3*-*dehydro-6-deoxoteasterone; 6*-*DeoxoTY, 6*-*deoxotyphasterol; 6*-*DeoxoCS, 6*-*deoxocastasterone; CT, cathasterone; TE, teasterone; TY, typhasterol; CS, castasterone; BL, brassinolide; ND, not detected. (d) qRT-PCR analysis of BR biosynthetic genes in Ws*-*2, *bri1-5*, *phyB-77* and *phyB-77 bri1-5* seedlings grown in red light for 5 days with a photon fluence rate of 30 μmol m^−2^ s^−1^ at 22°C. Error bars represent standard errors. Values were normalized to the expression of *UBQ10*.

To learn how BR signaling interacts with this *gul2/phyB*, we first examined the epistatic interaction between *phyB* and a weak *brassinosteroid insensitive 1* mutant *bri1-5* (Noguchi *et al.*, 1999). The phenotype of a double mutant of the strong allele *bri1-1* and *phyB-78* was indistinguishable from that of the *bri1-1* single mutant, whereas the weak allele *bri1-5* was dramatically suppressed by *phyB-77* mutation (Figure 1a,b), suggesting that BR signaling is essential for light-dependent growth. The elongated phenotype of the *phyB-77* single or *phyB-77 bri1-5* double mutant could stem from increased BR biosynthesis or signaling, or both. Therefore, we quantified endogenous BRs and noticed that the levels of the most bioactive BR, brassinolide (BL), were significantly lower in the *phyB-77 bri1-5* double mutant than in the *bri1-5* single mutant (Figure 1c). As a reduced level of BL is considered a consequence of increased BR signaling activity, mediated by BZR1 (Choe *et al.*, 2001; Wang *et al.*, 2002; Chung *et al.*, 2011, 2012; Kim *et al.*, 2013), the reduction in bioactive BR levels in *phyB-78* and *phyB-77 bri1-5* mutants implies that feedback downregulation signaling is activated by *phyB*. Consistently, the transcript levels of BR biosynthetic genes were low in *phyB-77* *bri1-5* mutant seedlings (Figure 1d). The transcript levels of *CONSTITUTIVE PHOTOMORPHOGENESIS AND DWARFISM* (*CPD*), *DWARF 4* (*DWF4*) and *CYP85A2* were high in *bri1-5*, but were suppressed in the double mutant, suggesting that feedback downregulation mechanisms are functional in the double mutant because of *phyB-77*.

**Figure 2:**
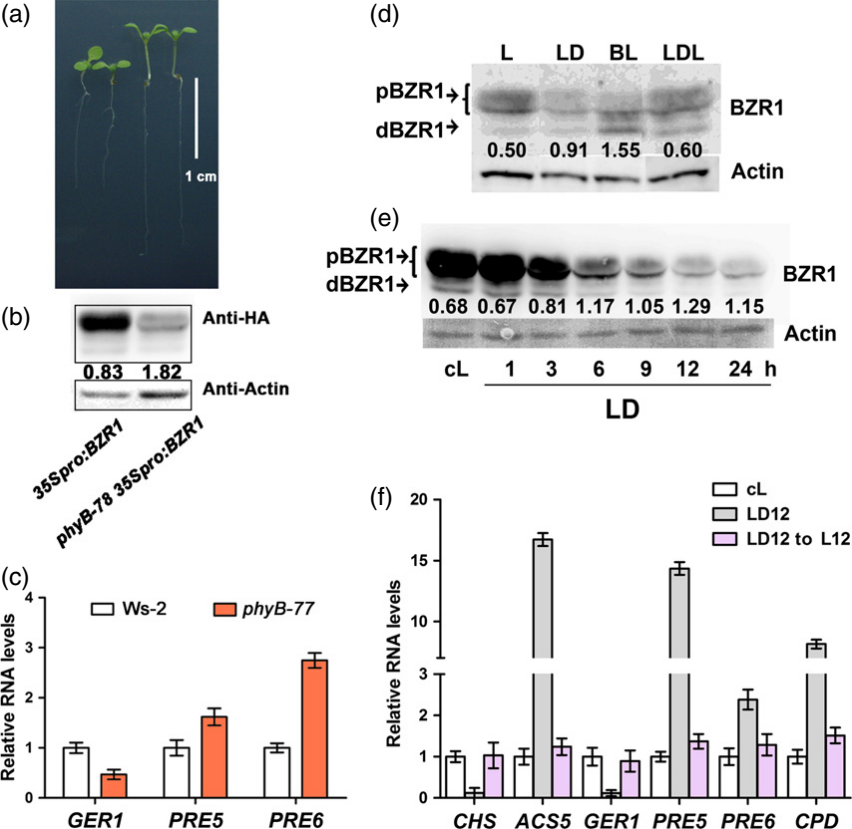
Brassinosteroid (BR) signal increases in the dark. (a) Phenotypes of 7*-*day-old *35Spro:BZR1-HA* and *phyB-78 35Spro:BZR1-HA* seedlings, and the corresponding (b) protein gel blot. The numbers indicate the ratio of dBZR1 to pBZR1. (c) qRT-PCR analysis of *GER1*, *PRE5* and *PRE6* in Ws*-*2 and *phyB-77* seedlings grown under red light for 5 days with a photon fluence rate of 80 μmol m^−2^ s^−1^ at 22°C. (d, e) Protein gel blots showing the abundance of the phosphorylated (pBZR1) and dephosphorylated (dBZR1) forms of BZR1 in different light conditions. (d) Seedlings (7 days old) expressing *BZR1pro:BZR1-HA* were subjected to various conditions: L, continuous light; LD, continuous light followed by transfer to dark for 6 h; BL, brassinolide treatment; LDL, transfer to darkness for 6 h and subsequently to light for 6 h with a photon fluence rate of 80 μmol m^−2^ s^−1^ at 22°C. (e) *35Spro:BZR1-HA* seedlings were grown in continuous light for 7 days with a photon fluence rate of 103 μmol m^−2^ s^−1^, and were transferred to darkness for 1–24 h, as indicated. In both panels, actin was used as an equal loading control and the numbers indicate the ratio of dBZR1 to pBZR1. (f) qRT-PCR analysis of the expression of *CHALCONE SYNTHASE* (*CHS*), *ACS5*, *GER1*, *PRE5*, *PRE6* and *CPD* in 10*-*day-old plants grown under continuous light (cL), light followed by darkness for 12 h (LD12) or light transferred to darkness for 12 h and back to light for 12 h (LD12 → L12). The photon fluence rate during light treatment was 103 μmol m^−2^ s^−1^. Error bars in (c) and (f) are standard deviations (*n* = 3).

To further examine the contribution of BZR1 in *phyB* signaling, we generated the *phyB-78* *35Spro:BZR1* line and determined the ratio of dephosphorylated BZR1 (dBZR1) to phosphorylated BZR1 (pBZR1; Figure 2a), a read-out of the BR signaling status (Wang *et al.*, 2002). The *phyB-78* *35Spro:BZR1* line exhibited a long hypocotyl phenotype relative to *35Spro:BZR1* transgenic seedlings (Figure 2a). Furthermore, the phosphorylated form of BZR1 was reduced in the *phyB-78* background (Figure 2b). Next, we examined the expression levels of genes known to be regulated by BZR1 in a light-dependent manner (Oh *et al.*, 2012): *GER1* was suppressed, and *PRE5* and *PRE6* were upregulated, in *phyB* mutants relative to the wild type (Figure 2c), suggesting that the elongated phenotype of *phyB* depends on the activation of BZR1 signaling.

### *phyB-77* activates BR signaling by reducing the abundance of BZR1 proteins

To identify the precise role of BZR1 in *phyB-77*, we sought to establish how light signaling modulates BR signaling pathways. As *phyB* mutants exhibits the same growth patterns as those of plants grown under shade, we performed the following experiments in total darkness or continuous white light (Figure 2d). After subjecting *BZR1pro:BZR1-HA* plants to various light conditions (L, light-grown control; LD, transferred to darkness for 6 h; LDL, transferred to darkness for 6 h and subsequently to light for 6 h), we found that most of the phosphorylated forms of BZR1 (pBZR1) disappeared in LD (Figure 2d), similar to the case under BL treatment (lane BL in Figure 2d), whereas the amount of protein in the dephosphorylated form (dBZR1) was similar for both LD and L (Figure 2d). The ratio of dBZR1 to pBZR1 increased upon both dark and BL treatment, indicating that BR signaling was activated by dark treatment. Conversely, when the dark-treated samples were returned to the light, pBZR1 became the dominant form (Figure 2d). Furthermore, a time-course analysis of a *35Spro:BZR1-HA* line confirmed the darkness-dependent removal of BZR1 (Figure 2e). BZR1 levels gradually decreased and the dBZR1/pBZR1 ratio increased from as early as 3 h after dark treatment (Figure 2e). In support of the effect of darkness on BZR1 activity, the expression of marker genes known to be activated by BZR1, such as of *ACC SYNTHASE 5* (*ACS5*), *PRE5*, *PRE6* and *CPD* (Oh *et al.*, 2012), was upregulated in the LD12 sample (light-grown seedlings transferred to darkness for 12 h before RNA isolation), and reverted to control levels upon return to light in the sample designated as ‘LD12 → L12’ (Figure 2f). In addition, genes known to be repressed by darkness were downregulated in LD (Figure 2f). This dark-specific removal of BZR1, preferentially the phosphorylated form, and the simultaneous regulation of known marker genes suggest that darkness activates BR signaling and hypocotyl growth by changing the abundance of BZR1.

### Regulation of BZR1 stability by COP1

**Figure 3:**
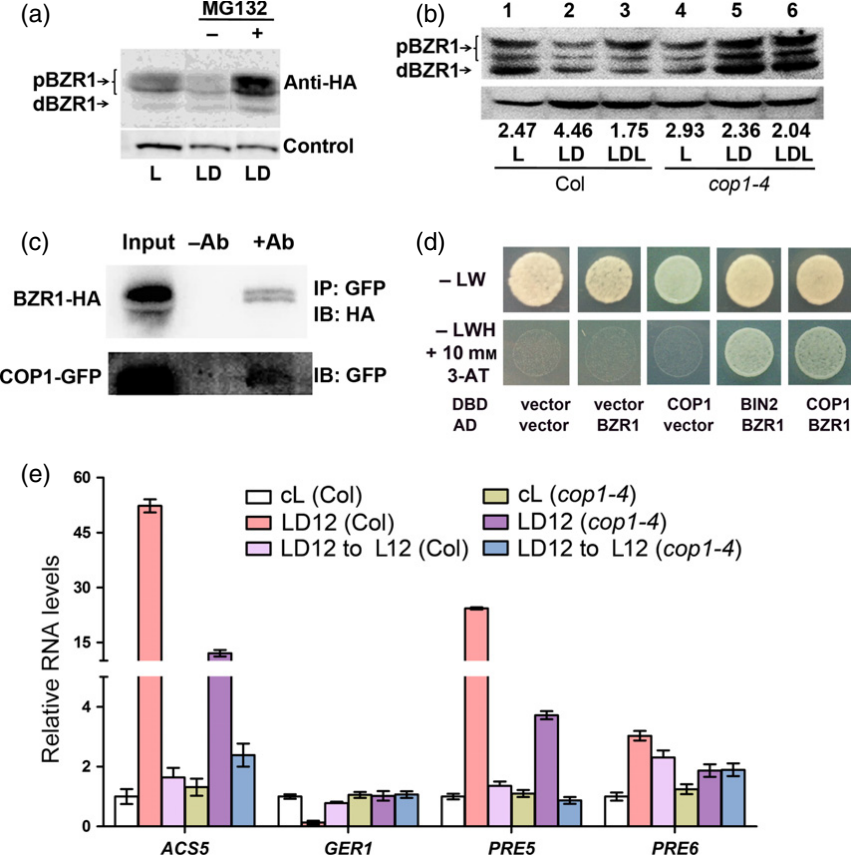
BZR1 stability is regulated by COP1 in a 26S proteasome-dependent manner. (a) Immunoblot analysis of BZR1 proteins. Seven-day-old light-grown seedlings expressing *BZR1pro:BZR1-HA* were treated or not with MG132 in the dark for 6 h. Actin was used as a loading control. (b) Immunoblot analysis results of the 10*-*day-old seedlings grown under continuous light and subsequently subjected to LD (light to dark for 12 h) or LDL (light to dark for 12 h and back to light for 12 h) growth conditions. The total protein extracted from 10-day-old seedlings was immunoblotted with anti-BZR1. Actin was used as a loading control. (c) Coimmunoprecipitation assay to test for the interaction between BZR1 and COP1 in *35Spro:BZR1-HA 35Spro:COP1-GFP* Arabidopsis seedlings. Seedlings were grown under long-day conditions (with a photon fluence rate of 103 μmol m^−2^ s^−1^), and then transferred to the dark for 2 days. Total proteins were first immunoprecipitated with anti-GFP, and were subsequently immunoblotted with anti-HA and anti-GFP. (d) Yeast two hybrid analysis. –LW indicates growth media lacking leucine (L) and tryptophan (W). DBD and AD are the vectors harboring the DNA binding domain and the DNA activation domain, respectively. Vector indicates the empty vector. (e) qRT-PCR analyses of *ACS5*, *GER1*, *PRE5* and *PRE6* in Col*-*0 and *cop1-4* treated with the same growth and light conditions as described in (b).

The dramatic removal of BZR1 upon dark treatment led us to speculate that BZR1 degradation is associated with a 26S proteasome. When light-grown seedlings were treated with both darkness and MG132, a 26S proteasome inhibitor, BZR1 noticeably accumulated (Figure 3a), suggesting that the 26S proteasome is involved in the removal of BZR1. Therefore, we next sought to identify an E3 ligase that binds to BZR1. We tested COP1, because it is known to be active in darkness (Osterlund *et al.*, 2000; Seo *et al.*, 2004). Compared with the Col*-*0 wild type (Figure 3b, lane 2), the *cop1-4* mutant accumulated BZR1 even after dark treatment (lane 5), suggesting that COP1 is involved in regulating BZR1 stability. An interaction between COP1 and BZR1 was further assessed through *in vivo* co-immunoprecipitation (CoIP) analyses (Figure 3c). Seedlings of double transgenic lines, *35Spro:BZR1-HA 35Spro:COP1-GFP*, were grown under long-day conditions, transferred to darkness and incubated for two more days. The total proteins were prepared and immunoprecipitated using anti-GFP antibody. When the precipitated proteins were separated and immunoblotted with anti-HA antibody, BZR1-HA proteins were detected (Figure 3c). CoIP data indicate that COP1 interacted with BZR1, especially pBZR1 (Figure 3c). A possible interaction between COP1 and BZR1 was further assessed through yeast two-hybrid analysis (Figure 3d). Similar to a positive control that involved the binding of BIN2 to BZR1 (He *et al.*, 2002), COP1 bound to BZR1 (Figure 3d). Both CoIP and the yeast two-hybrid test suggest that BZR1 and COP1 bind together as part of biological signal processing mechanisms.

We then reasoned that the expression patterns of the marker genes would be affected in the *cop1-4* mutant background. We monitored the expression of various marker genes in both wild-type and *cop1-4* seedlings grown in three different conditions: continuous light (cL); continuous light-grown seedlings transferred to darkness for 12 h (LD12); and LD12 seedlings transferred back to the light for 12 h (LD12 → L12). Compared with the wild-type control, the induction of *ACS5*, *PRE5* and *PRE6* by darkness was greatly suppressed in *cop1-4* mutants (Figure 3e). However, the darkness-dependent induction of these genes was canceled in the LD12 → L12 samples of both wild-type and *cop1-4* backgrounds, suggesting that light-dependent repression regularly occurs in *cop1-4* (Figure 3e). In support of this, we found that BZR1 accumulated in a similarly treated LDL sample of *cop1-4* mutant seedlings (Figure 3b). An oppositely regulated gene, *GER1*, was also misregulated in *cop1-4* (Figure 3e). Our CoIP results, coupled with the findings that BZR1 accumulates in *cop1-4* seedlings and that gene expression patterns are altered in *cop1-4,* suggest that binding of COP1 to BZR1 mediates preoteasome-dependent degradation, and that the uncoupling of BZR1 degradation from darkness-triggered signaling caused misexpression of the BZR1-regulated marker genes.

### Identification of genes co-regulated by both light and BR signaling

**Figure 4:**
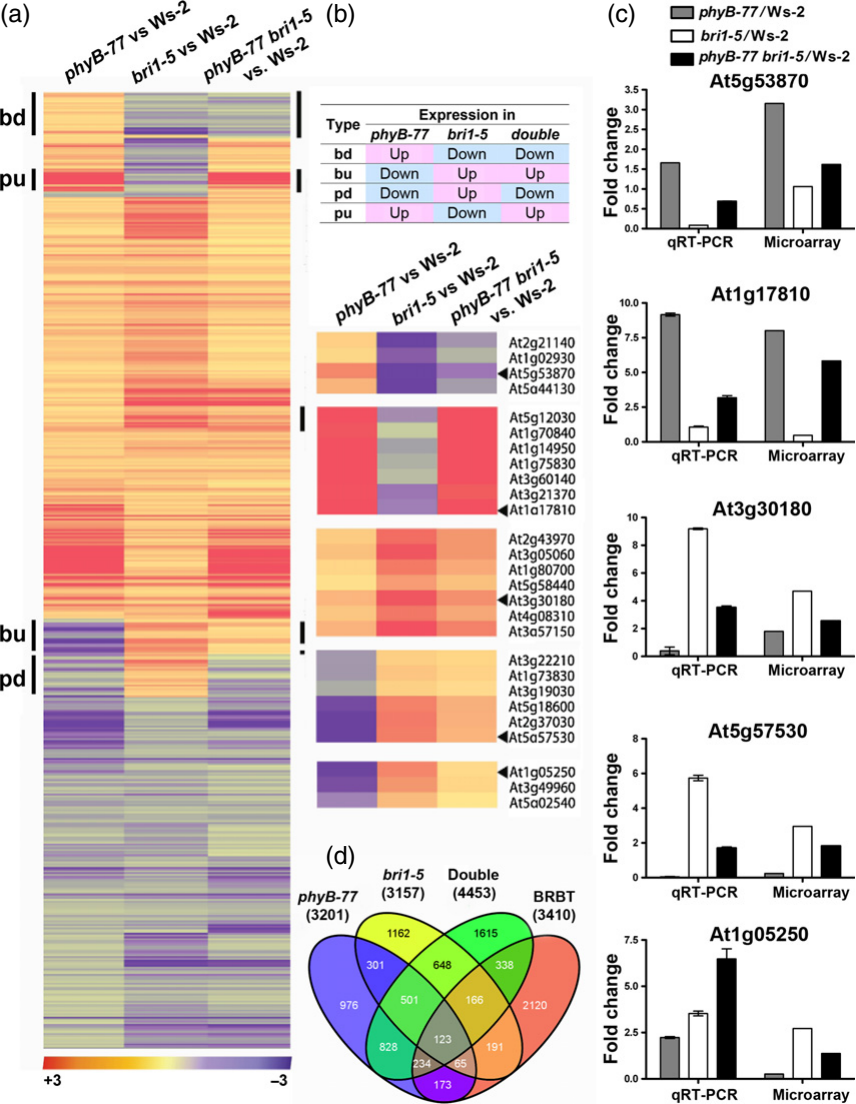
Identification of genes regulated by both light and brassinosteroids (BRs). (a, b) Heat maps of the 624 hierarchically clustered genes for which expression levels changed by more than 1.5-fold in *phyB-77*, *bri1-5* and *phyB-77 bri1-5*, relative to the Ws*-*2 wild type. Seedlings were grown in red light for 5 days with a photon fluence rate of 30 μmol m^−2^ s^−1^ at 22°C. Genes labeled as ‘bd’ at the left of the heat map were upregulated in *phyB-77* but downregulated in the *bri1-5* single and *phyB-77 bri1-5* double mutant, relative to the wild type. Genes downregulated in *phyB-77*, but upregulated in the *bri1-5* single and *phyB-77 bri1-5* double mutant were labeled ‘bu’. Genes designated as ‘pu’ were upregulated in *phyB-77* and the double mutant, but downregulated in *bri1-5*, and those named ‘pd’ were downregulated in *phyB-77* and the double mutant, but upregulated in *bri1-5*. (b) Definition of the gene clusters, and magnified view of the groups marked with vertical bars. (c) Validation of the microarray data using qRT-PCR. Error bars are the standard deviations (*n *= 3). (d) Venn diagram of the co-regulated genes in *phyB-77*, *bri1-5* and *phyB-77 bri1-5*, relative to Ws*-*2, and the set of genes defined as BZR1 binding target (BRBT) genes (Oh *et al.*, 2012).

To define the list of genes that are co-regulated by BRs and light, we performed a genome-wide transcriptome analysis using total RNAs prepared from the seedlings of four genotypes, i.e. Ws*-*2 wild type, *phyB-77*, *bri1-5* and *phyB-77 bri1-5* (Figure 4). We constructed a heat map based on 624 genes obtained after filtering with the criteria of *P* < 0.01 and fold change > 1.5 in the following three comparisons: *phyB-77* versus Ws*-*2; *bri1-5* versus Ws*-*2; and *phyB-77 bri1-5* versus Ws*-*2 (Figure 4a,b; Tables S1–S5). We used these comparisons because they enabled us to identify epistatic interactions. According to the expression patterns summarized in Figure 4(b), *bri1-5* is epistatic to *phyB-77* for both ‘bd’ (i.e.genes upregulated in *phyB-77* but downregulated in the *bri1-5* single and *phyB-77 bri1-5* double mutant, relative to the wild type) and ‘bu’ (i.e. upregulated in the *bri1-5* single and *phyB-77 bri1-5* double mutant), and *phyB-77* is epistatic to *bri1-5* for both the ‘pu’ (i.e. upregulated in *phyB-77* and the double mutant) and ‘pd’ (i.e. downregulated in *phyB-77* and the double mutant, but upregulated in *bri1-5*) types. To validate the microarray data, we subjected the genes marked by arrowheads (Figure 4b) to quantitative RT-PCR analysis. The two genes, *At5g53870* and *At1g17810*, encode early nodulin-like protein 1 (ENODL1) and a major intrinsic protein family water channel protein, respectively, and were upregulated in *phyB-77* but were repressed in *bri1-5*. In contrast, *At3g30180* and *At5g57530* encode CYP85A2 (BR6ox2) and xyloglucan endotransglycosylase 12 (XTH12), respectively, and were found to be repressed in *phyB-77* but induced in *bri1-5* in both microarray and qRT-PCR analyses (Figure 4c). In addition, a Venn diagram analysis revealed 123 genes at the intersection of *phyB-77*, *bri1-5*, *phyB-77 bri1-5* and the genes defined as BR-regulated BZR1 targets (BRBTs; Figure 4d; Oh *et al.*, 2012). We postulate that the growth pattern regulated by light and BRs depends on complex interactions between these genes (Table S6).

### Functional validation of the identified genes

**Figure 5:**
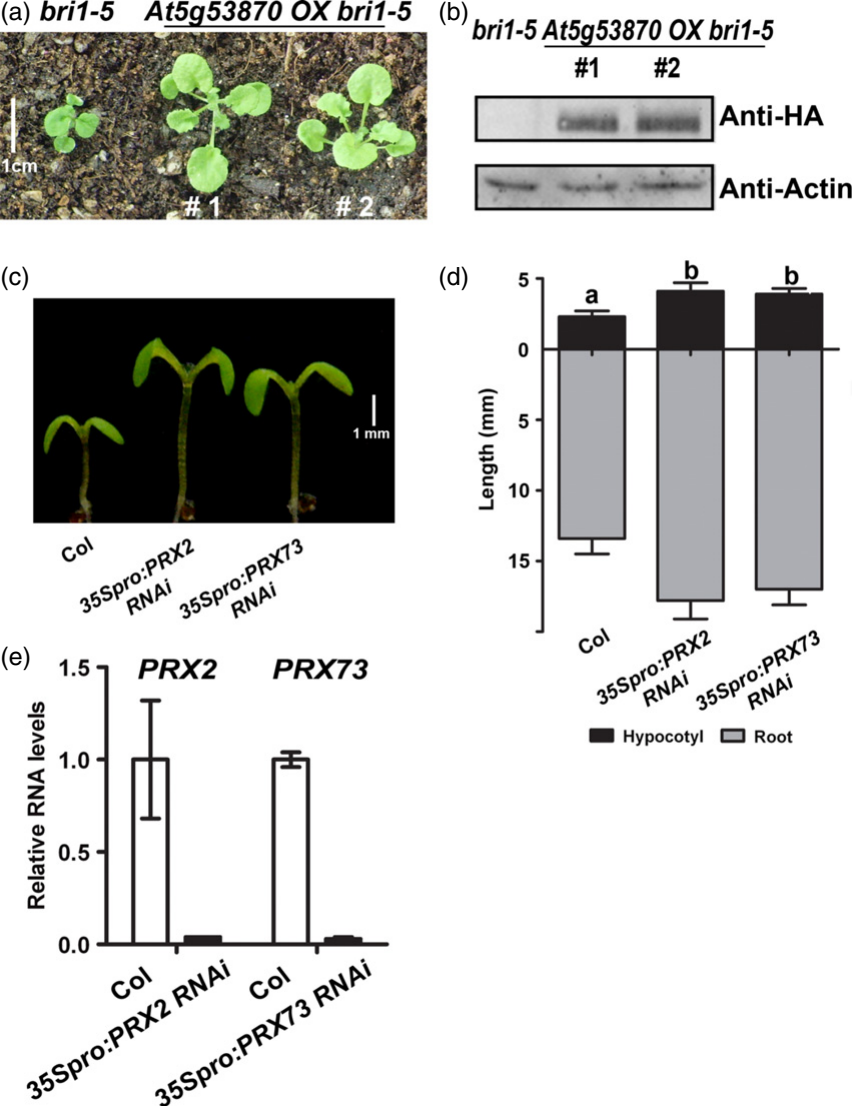
Knock-in of ENODL1 (At5g53870) and knock-down of peroxidases stimulates growth. (a, b) Phenotypes and protein levels of *bri1-5* plants and two transgenic lines overexpressing At5g53870 in the *bri1-5* background. (a) Comparison of 4*-*week-old plants exhibiting the partial suppression of *bri1-5*. (b) Immunoblot analysis showing At5g53870 expression in transgenic lines, compared with the *bri105* control. Proteins were extracted from the aerial tissues of adult plants. (c) *35Spro:PRX2 RNAi* and *35Spro:PRX73 RNAi* seedlings display elongated hypocotyls. (d) Measurement of hypocotyl and root lengths. Seedlings were grown on MS medium for 7 days under long-day conditions (16-h light/8*-*h dark, with a photon fluence rate of 80 μmol m^−2^ s^−1^ at 22°C. Error bars are standard deviations (*n* = 20; *P *< 0.001). Different lowercase letters above each bar indicate statistically significant differences. (e) Relative RNA levels of nascent *PRX2* and *PRX73* in wild-type and transgenic lines expressing *PRX RNAi*. Error bars are standard deviations (*n* = 3; *P *< 0.001).

To understand the effects of these genes on growth, the *35Spro:At5g53870-HA* construct was introduced into the *bri1-5* mutant background (Figure 5a,b). We found that the dwarf phenotype of *bri1-5* was suppressed, such that petioles elongated and leaves expanded (Figure 5a). The expression of *At5g53870* was confirmed by protein gel blot analysis (Figure 5b). Furthermore, we expressed two *PEROXIDASE*s (*PRX2* and *PRX73*) that were downregulated in *phyB-77* but were upregulated in *bri1-5* in the antisense orientation; both *PRX2* RNAi and *PRX73* RNAi transgenic plants exhibited elongated hypocotyls (Figure 5c,d), and the expression of the target genes was greatly reduced (Figure 5e).

### Formation of dimers between phosphorylated and dephosphorylated forms of BZR1

**Figure 6:**
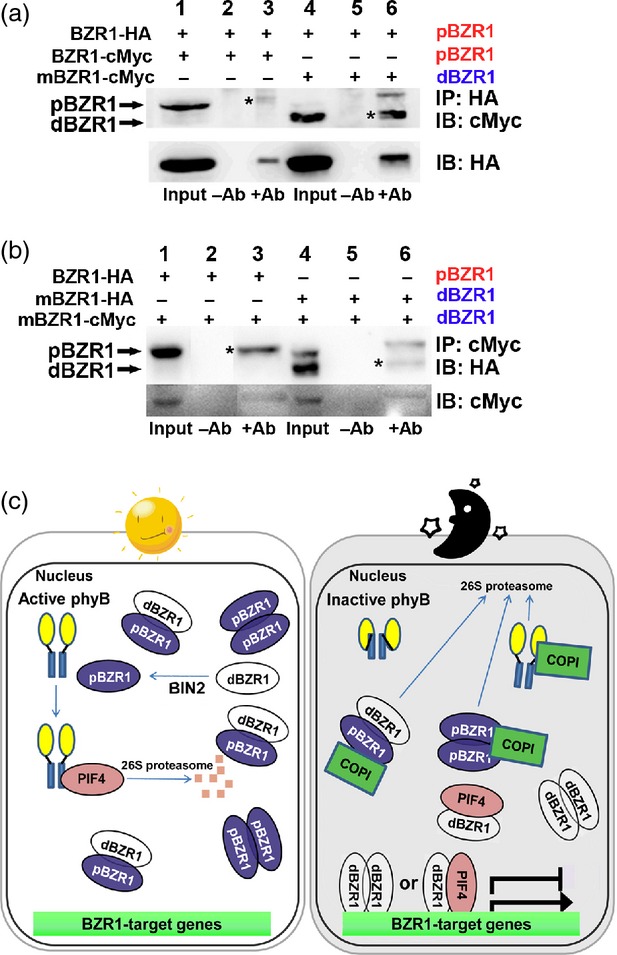
Different forms of BZR1 bind with each other as part of a regulatory mechanism. (a, b) Coimmunoprecipitation analysis showing the interaction between pBZR1 and dBZR1. Three-week-old Arabidopsis plants were used to isolate mesophyll-derived protoplasts that were transfected with *35Spro:BZR1-HA* and *35Spro:BZR1-cMyc* variants. Plants were grown under long-day conditions (16-h light/8-h dark, with a photon fluence rate of 80 μmol m^−2^ s^−1^ at 22°C. Protein was immunoprecipited with anti-HA (a) or anti-cMyc (b) antibody. mBZR1-HA and mBZR1-cMyc indicate BZR1^S130A^-HA and BZR1^S130A/134A^-cMyc, respectively. Protein bands with asterisks are compared for intensity. (c) Working model of the interaction between light and the brassinosteroid signaling pathways.

Next, we sought to explain the darkness-dependent degradation of pBZR in terms of BR signaling. We hypothesized that BZR1 proteins with different levels of phosphorylation may bind with each other as part of a regulatory mechanism. To demonstrate binding between BZR1 proteins, we transiently expressed the epitope-tagged BZR1 constructs in protoplasts and carried out a CoIP analysis (Figure 6a,b). In our transient expression system, *35Spro:BZR1-HA* and *35Spro:BZR1-cMyc* constructs produced phosphorylated forms of BZR1 (Figure 6a, lane 1). The *35Spro:mBZR1-HA* and *35Spro:mBZR1-cMyc* constructs were previously reported to have mutations of S130A and S130A/S134A, respectively, in BZR1 that reduced the levels of phosphorylation (Ryu *et al.*, 2007). We were able to clearly discern the proteins derived from these constructs as phosphorylated (shifted toward the top of the gel; Figure 6a, lanes 1 and 4) or dephosphorylated (Figure 6b, lanes 1 and 4) forms.

Lane 3 in Figure 6a shows the pBZR1-cMyc protein derived from a complex of pBZR1-cMyc and pBZR1-HA, and lane 6 indicates dBZR1-cMyc derived from the pBZR1-HA and dBZR1-cMyc complex (asterisk). This CoIP result suggests that BZR1 proteins with different levels of phosphorylation can form dimers with each other. To avoid a possible bias derived from the epitopes, and to determine the affinity of dBZR1 for dBZR1, we repeated the pull-down assay (Figure 6b). The pBZR1-HA derived from the pBZR-HA and dBZR1-cMyc complex (lane 3), and the dBZR-HA proteins from the dBZR-HA and dBZR1-cMyc complex (lane 6), were clearly detected. Interestingly, it seemed that pBZR-HA and dBZR1-cMyc (lane 3) had a greater affinity for each other than did dBZR-HA and dBZR1-cMyc (lane 6). The results of our pull-down assays suggest that BZR1 proteins can form dimers, and that pBZR1-pBZR1, pBZR1-dBZR1 and dBZR1-dBZR1 pairings are possible.

In conclusion, we propose a model that explains the roles of BZR1 in light and dark conditions. In the light (Figure 6c, left panel), the majority of BZR1 is phosphorylated by BIN2 and remains inactive in the cytoplasm (Ryu *et al.*, 2007). Our results show that pBZR1 can trap dBZR1 to form a dimer between pBZR1 and dBZR1. In addition, PIF4 is labile in the light and less likely to form an active heterodimer with dBZR1. Because COP1 is not active in the nucleus in the light, the pools of pBZR1 and dBZR1 are maintained; however, in darkness (Figure 6c, right panel), COP1 becomes active and preferentially targets BZR1 for degradation, because pBZR1 is the major form present. The dBZR1 proteins, being freed from the complex of pBZR1 and dBZR1, can now form dimers with either dBZR1 or PIF4 to actively control downstream genes. It was previously shown that PIF4 can form a dimer with BZR1 and that the dimer controls the target genes that are regulated by both light and BRs (Oh *et al.*, 2012).

In addition, GA is known to modulate BR signaling through controlling the stability of DELLA proteins (Bai *et al.*, 2012). In darkness, the endogenous GA level goes up (Bae and Choi, 2008), resulting in the clearance of DELLA proteins (Itoh *et al.*, 2003; Wang and Deng, 2011). Signaled by this increased GA level, both BZR1 and PIF proteins are released from DELLA, which enables these two proteins to form an active heterodimer and regulate the genes involved in hypocotyl growth (Feng *et al.*, 2008; de Lucas *et al.*, 2008; Bai *et al.*, 2012; Jaillais and Vert, 2012). Our results fit well with this model. COP1-mediated degradation of pBZR1 should increase the possibility of dBZR1 to meet either dBZR1 or PIF4. The concerted action of GA and BR signals explains the mechanisms of hypocotyl growth under different light conditions. It is expected that future studies will reveal whether DELLA preferentially binds to pBZR1, and whether this binding affects the stability of pBZR1 protein in light.

We thus revealed a mechanism by which Arabidopsis seedlings can grow faster in darkness without a simultaneous increase in the level of bioactive BRs. Light conditions regulate the stability of BZR1 via COP1 E3 ligase to control the formation of active dimers of dBZR1-dBZR1 or dBZR1-PIF4.

## EXPERIMENTAL PROCEDURES

### Plant materials and growth conditions

Arabidopsis seeds were surface sterilized and plated on agar-solidified MS media. After stratification at 4°C for 3 days, seedlings were grown under long-day (16*-*h light/8*-*h dark), continuous red light or continuous white light conditions at 22°C.

To introduce *35Spro:ENODL1* (*At5g53870*) into *bri1-5*, and *35pro:COP1-GFP*, *35Spro:BZR1-HA* and *35Spro:PRX RNAi* constructs into wild-type plants, coding sequences of each gene were cloned into pENTR/SD/D_TOPO (Invitrogen, now Life Technologies, http://www.lifetechnologies.com), and then combined with the destination vector pEarley101, pMDC83 and pB7GWIWGII in frame with epitopes, respectively. The constructs were transformed into Arabidopsis using conventional techniques.

### Quantitative analysis of endogenous BRs and sterols by gas chromatography–mass spectrometry (GC-MS)

To determine the endogenous levels of both sterols and BRs, the aerial parts of 5*-*week-old Arabidopsis plants were harvested to yield approximately 30 g of fresh tissues, and then lyophilized at –80°C. The tissues were extracted twice with 300 ml of methanol. Deuterium-labeled internal standards were added to the extracts. Purification and quantification of sterols and BRs were performed according to a method described previously (Fujioka *et al.*, 2002).

### Coimmunoprecipitation

Previously, it was shown that site-directed substitution of Ser130 alone, or of both Ser130 and Ser134, with Ala in BZR1, designated as mBZR1-HA^S130A^ and mBZR1-cMyc^S130,134A^, respectively, exhibited lower levels of phosphorylation than a wild-type form (Ryu *et al.*, 2007). These mutated versions were used to represent dephosphorylated forms. The plasmid DNA constructs were transfected into leaf mesophyll-derived protoplasts that were freshly isolated from 3–week-old wild-type plants as previously described (Hwang and Sheen, 2001). Briefly, approximately 2 × 10^5^ protoplasts were transfected with 30 μg BZR1 and mBZR1 plasmid DNA. Transfected protoplasts were incubated for 6 h at room temperature and homogenized in immunoprecipitation buffer containing 50 mm Tris–HCl (pH 7.5), 100 mm NaCl, 2 mm DTT, 0.1% Triton X*-*100, and full strength (1X) protease inhibitor cocktail (GenDEPOT, http://www.gendepot.com). The supernatant was incubated for 3 h at 4°C with agarose-conjugated anti-HA (Roche, http://www.roche.com) and then washed three times with dH_2_O. Then, 30 μl of denaturated proteins was subjected to SDS-PAGE on a 10% gel. After transfer to polyvinylidene difluoride membranes, the blot was detected with anti-cMyc (Cell Signaling Technology, http://www.cellsignal.com) or anti-HA (Roche) diluted at 1/2000. The immunoblot was washed with phosphate-buffered saline buffer and incubated with the corresponding anti-horseradish peroxidase (HRP) antibodies for visualization of the protein bands (GenDEPOT).

### Yeast two-hybrid assays

Full-length coding sequences of BZR1, BIN2 and COP1 were cloned into the low-copy yeast expression vectors pDEST22 (Gal4 AD) and pDEST32 (Gal4 BD). To test protein interactions, the corresponding plasmids were co-transformed into the yeast MaV203 strain. Successfully transformed colonies were identified on yeast synthetic drop-out media that lacked Leu and Trp (Clontech, http://www.clontech.com). At 3 days after transformation, yeast colonies were grown in selective –Leu, –Trp liquid media for 24 h, and the cell density was adjusted to OD_600_ = 0.1. An ∼10*-*μl volume of cell suspension was pipetted onto yeast synthetic drop-out media lacking His, Leu and Trp, and supplemented with 10 mM 3*-*amino-1,2,4-triazole (3*-*AT; Sigma-Aldrich, http://www.sigmaaldrich.com). Plates were incubated at 28°C for 2–4 days. The empty vectors pDEST22 and pDEST32 were co-transformed as negative controls.

### Protein extraction and immunoblot analysis

Total proteins were extracted from the tissues of 7*-*day-old seedlings grown on agar-solidified media using protein extraction buffer [50 mm Tris–Cl (pH 7.5), 100 mm NaCl, 10 mm MgCl_2_, 1 mm EDTA, 10% glycerol, 1 mm DTT, 1 mm phenylmethylsulfonyl fluoride and 1X protease inhibitor cocktail (GenDEPOT)]. Proteins were equally loaded on an 8 or 10% SDS–polyacrylamide gel and transferred to a polyvinylidene difluoride membrane. The antibodies used (at 1:3000) were anti-actin (MP Biomedicals, http://www.mpbio.com) and anti-BZR1 (generated in our lab). Following three washings with Tris-buffered saline Tween-20 (TBST) buffer, membranes were incubated with HRP-linked secondary antibodies (GenDEPOT). The membranes were then washed again with TBST, and antibodies were visualized with a chemiluminescence method (Amersham Bioscience, now GE Healthcare, http://www.gelifesciences.com). Protein gel blot analyses were performed multiple times, and the data shown are representative blots.

### Microarray and quantitative RT-PCR analysis

Microarray experiments were performed using an Affymetrix ATH1 GeneChip system. Seedlings of four genotypes, Ws*-*2 wild type, *bri1-5*, *phyB-77* and *bri1-5 phyB-77*, were grown for 7 days on 1X MS agar-solidified media under continuous red light. Biological samples were analyzed in triplicate and on three chips per genotype. The microarray analysis generated 12 different CEL files. All data generated during this experiment were submitted to the Gene Expression Omnibus (GEO) (http://www.ncbi.nlm.nih.gov/projects/geo/) under accession number GSE46456. Statistical analyses of the data were performed according to methods described previously (Chung *et al.*, 2011).

For validation of the microarray data, 7*-*day-old light-grown seedlings were ground to a fine powder in liquid nitrogen. Total RNA was prepared using Trizol (Sigma-Aldrich). Two micrograms of total RNA was reverse-transcribed (Fermentas, now Thermo Scientific, http://www.thermoscientificbio.com/fermentas), and equal quantities of reverse-transcribed products were used for the PCR reactions. Each template RNA was normalized using the *UBQ10* (*AT4G05320*) or *TUB2* (*AT5G62690*) genes as loading controls. Quantitative RT-PCR was performed in 96*-*well plates (Applied Biosystems, http://www.appliedbiosystems.com) using a commercial qPCR kit (KapaBiosystems, http://www.kapabiosystems.com) in a volume of 20 μl. The reactions were performed in triplicate. Samples were quantified by generating standard curves using the results obtained with serially diluted control DNA. The relative mRNA levels represent fold change over the control. Each cDNA was normalized using *UBQ10* or *TUB2*. The oligonucleotide sequences used in the qRT-PCR are listed in Table S7.

## SUPPORTING INFORMATION

Table S1 Complete data of the microarray analysis using the RNAs prepared from Ws–2 wild type, *phyB-77*, *bri1-5* and *phyB-77 bri1-5* seedlings grown for 7 days under continuous red light.

Table S2 Expression data for 3201 genes, with a comparison of *phyB-77* versus Ws*-*2 (*P *< 0.01).

Table S3 Expression data for 3157 genes, with a comparison of *bri1-5* versus Ws*-*2 (*P *< 0.01).

Table S4 Expression data for 4453 genes, with a comparison of *phyB-77 bri1-5* versus Ws*-*2 (p < 0.01).

Table S5 Expression data for 624 genes, with *P *< 0.01 at all three comparisons of *phyB-77* versus Ws*-*2, *bri1-5* versus Ws*-*2 and *phyB-77 bri1-5* versus Ws*-*2.

Table S6 Expression data for the genes at each intersection in the Venn diagram.

Table S7 List of primers used in quantitative RT-PCR and cloning.
