# Supplementary material for: Darkness and gulliver2/phyB mutation decrease the abundance of phosphorylated BZR1 to activate brassinosteroid signaling in Arabidopsis
Source: Plant J. 2014 Feb 4;77(5):737–47. doi: 10.1111/tpj.12423 (PMC4282538; doi:10.1111/tpj.12423)
Supplement: Table S7 — List of primers used in quantitative RT-PCR and cloning. [file tpj0077-0737-SD7.docx]

Table S7. List of primers used in quantitative RT-PCR and cloning.

| **Gene** | |  |  | **Primer sequences (5’ to 3’)** |
| --- | --- | --- | --- | --- |
|  | **For qRT-PCR** | | | |
| *CYP85A2*  *AT3G30180* | |  | F | AACGAATTACCGCAGTGGAG |
|  |  |  | R | AAGAGCCATCATGGAGGTTG |
| *DWF4*  *AT3G50660* | |  | F | GTT GGC CAT TTC TTG GTG AAA |
|  |  |  | R | TGG CGG TGT ACG GTT TAA GAT |
| *CPD*  *AT5G05690* | |  | F | ATGAAAAGGAGGGAGGAGGA |
|  |  |  | R | TGATCGTGGAGGTTGTTTCA |
| *UBQ10*  *AT4G05320* | |  | F | CCACCAAAGTTTTACATGAAACGAA |
|  |  |  | R | TCCAGGACAAGGAAGGTATTCC |
| *TUB2* *AT5G62690* | |  | F | ATCCGTGAAGAGTACCCAGAT |
|  |  |  | R | AAGAACCATGCACTCATCAGC |
| *PP2A* *AT1G13320* | |  | F | TATCGGATGACGATTCTTCGTGCAG |
|  |  |  | R | GCTTGGTCGACTATCGGAATGAGAG |
| *ACS5 AT5G65800* | |  | F | GCGATGCTTTCCTTTTGCCTACTC |
|  |  |  | R | TTTCTGGGCTTGTTGGTAAGCTTGT |
| *PRE5* *AT3G28857* | |  | F | AACGGCGTCGTTCTGATAAG |
|  |  |  | R | CATGAGTAAGCTTCTAATCACGG |
| *PRE6*  *AT1G26945* | |  | F | TCCAACACCTCATCCCTGAACTTCG |
|  |  |  | R | CGGTCACTGAGGTCATCAACCTCTC |
| At1g05250 | |  | F | AGGTCGTGGCTCAGAAGAAA |
|  |  |  | R | TTGCCGGTAAGAATTTGGAC |
| *GER1*  *AT1G72610* | |  | F | CCTGCGGGTTACCCTTGCATTC |
|  |  |  | R | AGCGGCGTTGATGATGTTTGTAGTG |
| At5g53870 | |  | F | GCGTCTGCTCCTTCAAAATC |
|  |  |  | R | ATTTTGGTGACGGAGATGGA |
| At5g57530 | |  | F | TGTTGTTGGCGTCTCTTCTG |
|  |  |  | R | AACCTGAACCGGATGTCTTG |
| At1g17810 | |  | F | GACTCACCCGGACTCCATTA |
|  |  |  | R | CCACCAGAACTAACCCTCCA |
| *PRX2*  *AT1G05250* | |  | F | CGAAATTGAACGATGCATTGCTAAA |
|  |  |  | R | CCATGTTCAGTGAGGTTCTGAAAT |
| *PRX73*  *AT5G67400* | |  | F | CCGGACCAAATAACAAAGTTACAGAA |
|  |  |  | R | GCGGTTACGAAAGCCTTGTTGAAA |
|  | **For cloning** | | | |
| At5g53870 | |  | F | TGTTGTTGGCGTCTCTTCTG |
|  |  |  | R | AACCTGAACCGGATGTCTTG |
| *COP1* *AT2G32950* | |  | F | GACTCACCCGGACTCCATTA |
|  |  |  | R | CCACCAGAACTAACCCTCCA |
| *BIN2 AT4G18710* | |  | F | GGATCCTAAAATGGACAGCAAACTGA |
|  |  |  | R | AAATCTGACACAATCGACCTGGTTGAT |
| *BZR1 AT1G75080* | |  | F | CGAAGGGAACTTACCAGGACCTT |
|  |  |  | R | TTGTCAAACGTCTTGGGCGTGAC |
| *PRX2* *AT1G05250* | |  | F | CACCATGGCGATCAAGAACATTCTCGC |
|  |  |  | R | GTTAGGGAAGGCGCATCTCTTC |
| *PRX73*  *AT5G67400* | |  | F | CACCATGGCGCGGTTCAGTCTGGTT |
|  |  |  | R | GTTAAAGGCACCACAGTCACGAC |
