# Supplementary material for: Darkness and gulliver2/phyB mutation decrease the abundance of phosphorylated BZR1 to activate brassinosteroid signaling in Arabidopsis
Source: Plant J. 2014 Feb 4;77(5):737–47. doi: 10.1111/tpj.12423 (PMC4282538; doi:10.1111/tpj.12423)
Supplement: Table S7 — List of primers used in quantitative RT-PCR and cloning. [file tpj0077-0737-SD8.docx]

Table S1. Complete data of the microarray analysis using the RNAs prepared from Ws-2 wild type, *phyB-77*, *bri1-5*, and *phyB-77 bri1-5* seedlings grown for 7 days under continuous red light.

Table S2. Expression data for 3201 genes with the comparison of *phyB-77* vs. Ws-2. The list of genes were obtained after filtering with the criteria of p-value < 0.01 and fold change > 1.5.

Table S3. Expression data for 3157 genes with the comparison of *bri1-5* vs. Ws-2 (p<0.01).

Table S4. Expression data for 4453 genes with the comparison of *phyB-77 bri1-5* vs. Ws-2 (p<0.01).

Table S5. Expression data for 624 genes with p<0.01 at all three comparisons of *phyB-77* vs. Ws-2; *bri1-5* vs. *Ws-2*; and *phyB-77* *bri1-5* vs. Ws-2.

Table S6. Expression data for the genes of each intersection in the Venn diagram.

Table S7. List of primers used in quantitative RT-PCR and cloning.
